# Multi-Functional Core-Shell Nanofibers for Wound Healing

**DOI:** 10.3390/nano11061546

**Published:** 2021-06-11

**Authors:** Zhen Li, Shunqi Mei, Yajie Dong, Fenghua She, Puwang Li, Yongzhen Li, Lingxue Kong

**Affiliations:** 1 Hubei Key Laboratory of Digital Textile Equipment, Wuhan Textile University, Wuhan 430073, China; zkj@deakin.edu.au (Z.L.); dongy@deakin.edu.au (Y.D.); 2 Institute for Frontier Materials, Deakin University, Geelong, VIC 3216, Australia; mary.she@deakin.edu.au; 3 Foshan Green Intelligent Manufacturing Research Institute of Xiangtan University, Foshan 528000, China; 4 South Subtropical Crop Research Institute, Chinese Academy of Tropical Agricultural Sciences, Zhanjiang 524091, China; puwangli@catas.cn; 5 Agricultural Product Processing Research Institute, Chinese Academy of Tropical Agricultural Sciences, Zhanjiang 524001, China; liyongzhen@catas.cn

**Keywords:** core-shell nanofiber, drug delivery, co-axial centrifugal spinning, wound healing

## Abstract

Core-shell nanofibers have great potential for bio-medical applications such as wound healing dressings where multiple drugs and growth factors are expected to be delivered at different healing phases. Compared to monoaxial nanofibers, core-shell nanofibers can control the drug release profile easier, providing sustainable and effective drugs and growth factors for wound healing. However, it is challenging to produce core-shell structured nanofibers with a high production rate at low energy consumption. Co-axial centrifugal spinning is an alternative method to address the above limitations to produce core-shell nanofibers effectively. In this study, a co-axial centrifugal spinning device was designed and assembled to produce core-shell nanofibers for controlling the release rate of ibuprofen and hEGF in inflammation and proliferation phases during the wound healing process. Core-shell structured nanofibers were confirmed by TEM. This work demonstrated that the co-axial centrifugal spinning is a high productivity process that can produce materials with a 3D environment mimicking natural tissue scaffold, and the specific drug can be loaded into different layers to control the drug release rate to improve the drug efficiency and promote wound healing.

## 1. Introduction

Polymeric nanofiber materials have been extensively studied for various bio-medical applications including tissue engineering [1,2], wound healing [3,4], and drug delivery [5,6]. Generally, those nanofibers are monoaxial structured [7,8,9,10], though core-shell structured nanofibers functionalized with bioactive molecules [6,11,12] are more suitable for drug delivery in wound healing. For example, the shell layer of the core-shell nanofibers could not only have an anti-infection property, but also avoid drugs or bioactive agents in the core layer released immediately and prevent bioactive agents from losing bioactivity in the wound environment [13,14]. In addition, the release rate of the drug encapsulated in the core layer can be well controlled [6] by designing an appropriate shell layer. Coaxial electrospinning is the main method for core-shell nanofiber fabrication but its production rate is low and energy consumption is high [15,16,17]. Centrifugal spinning is an emerging approach to fabricating core-shell nanofibers with high-efficiency and at low cost [18].

Centrifugal spinning is a technology that uses high rotation speed to fabricate three-dimensional (3D) structured nanofibers [16,19,20]. In principle, centrifugal spinning utilizes high rotational speed to generate centrifugal force to overcome the surface tension of the polymer solution and eject the polymer solution from the reservoir into the air. The jet is then stretched by forces and the solvent evaporates during the whole process. Finally, the fibers are collected on a collector. The process of centrifugal spinning can be divided into three stages: jet-initiating, jet-extension and solvent evaporation stages [19]. Centrifugal spinning has been used to produce core-shell nanofibers for tissue regeneration [21] and drug delivery [22]. 

Wound healing is a complex and multifactorial process, often referred to as the cascade of healing including four phases: hemostasis, inflammation, proliferation, and remodeling [23]. These phases are an integrated and synchronized process, depending on various mediators of the extracellular matrix (ECM), cell growth factors, platelets, cytokines, and chemokines, among others [24,25,26]. Ideal wound healing dressing is expected to be multifunctional and able to deliver different drugs needed at different healing stages. Chitosan is a renewable biodegradable polymer with antibacterial ability and hemostasis property and therefore has been widely used as a natural ECM for wound healing [27,28,29,30]. However, native chitosan has to be solved into some toxic or acidic solutions such as chloroform [24] and acetic acid [31] and the residual solvent could be released during the delivery [32]. Carboxylated chitosan (CCS) could be used for wound healing activities since its water-soluble property can fulfill chitosan’s potentials for wound healing applications, rather than being restricted by toxic or acidic solvents [33]. Ibuprofen is a nonsteroidal drug with antibacterial and analgesic properties [34] and has been used for biomedical applications [31]. Besides, polyethylene oxide (PEO) is also a biodegradable polymer, which is usually blended with chitosan to fabricate chitosan composite nanofibers for bio-medical applications [4,35,36], as it can be utilized for improving the dimensional stability of the nanofiber structure [37].

Cell growth factors including epidermal growth factors (EGF), vascular endothelial growth factor (VEGF), transforming growth factor-β (TGF-β), and platelet-derived growth factor (PDGF) play a significant role in connecting cells and ECM to improve functional restoration of tissues at the proliferation phase [38]. For example, EGF is a critical mediator for keratinocytes to increase their proliferation and migration to improve skin regeneration and significantly reduce scar formation [39]. Currently, sustained delivery of growth factors on wounds is a big challenge as they can be chemically conjugated on the surface of nanofibers and lose their activity rapidly in the wound circumstance. Releasing growth factors by encapsulation is a non-controllable method as they tend to release explosively at the initial phase rather than the later phase (i.e., proliferation phase) [40].

In this paper, a novel biomimetic system was designed via co-axial centrifugal spinning technology to produce core-shell nanofibers for effective drug delivery and wound healing. Formation of core-shell structured nanofibers via self-assembled co-axial centrifugal spinning was investigated by changing various parameters. Two model drugs used as anti-inflammatory drug (ibuprofen) and human epidermal growth factors (hEGF) were loaded into the shell layer and core layer, respectively, to improve the multi-functionality of core-shell nanofibers for wound healing. The advantages of multi-functional core-shell nanofibers over monoaxial nanofibers and a commercial wound dressing (AquacelAg) were evaluated via drug release profiling, antibacterial assessment, and the cell viability assay. This work demonstrated that the drug release rate in core-shell nanofibers can be better controlled and different drugs can be delivered at different phases of wound healing. In addition, biodegradable water-soluble nanofibers with drugs or growth factors might be more suitable for wound healing compared with AquacelAg (hydrophilic fibrous wound dressing) as the secondary damage to the wound caused by any residual dressing during dressing change can be avoided.

## 2. Materials and Methods

### 2.1. Materials

Carboxylated chitosan (CCS, low molecular weight), polyethylene oxide (PEO, average Mw~1,000,000), and ibuprofen were purchased from Aladdin (Shanghai, China). Human epidermal growth factor (hEGF) was purchased from Sigma (St. Louis, MO, USA). *Escherichia coli* (*E. coli*), *Pseudomonas aeruginosa* (*P. aeruginosa*), and *Staphylococcus aureus* (*S. aureus*) were purchased from the Guangdong Microbial Culture Center (Guangzhou, China). A human skin fibroblast cell line (HSF, CRL-2522) was purchased from iCell Bioscience Inc. (Shanghai, China). AquacelAg was purchased from ConvaTec Inc. (Flintshire, UK). All materials were used as received without further modifications.

### 2.2. Fabrication of Nanofibers via Centrifugal Spinning

#### 2.2.1. Fabrication of Nanofibers without Drugs via Co-Axial Centrifugal Spinning

CCS powder was dissolved into deionized (DI) water at a concentration of 14 *w*/*v*%; PEO powder was dissolved into DI water at concentrations of 6 *w*/*v*% and 7 *w*/*v*%; CCS (14 *w*/*v*%) and PEO (7 *w*/*v*%) solutions were then mixed to prepare CCS/PEO composite solutions at 1:1 CCS/PEO and 2:1 CCS/PEO. All of the above solutions were stirred for 8 h at room temperature. Various parameters were changed to produce nanofibers via co-axial centrifugal spinning including viscosity, rotational speed, nozzle diameter, and solution flow ratio between core and shell layers. The collection distance was 30 cm and the temperature was 25 °C.

#### 2.2.2. Fabrication of Monoaxial and Core-Shell Nanofibers with Drugs

To prepare monoaxial nanofibers with two model drugs, 10 mg/mL ibuprofen and 1 μg/mL hEGF were mixed into a 1:1 CCS/PEO solution, followed by stirring for 15 min in an ice bath. The operational conditions of the centrifugal spinning system were: rotational speed of 4500 rpm, collection distance of 30 cm, and temperature of 25 °C. 

To prepare core-shell nanofibers with drugs, drug loading in each unit length for each drug in the core-shell nanofibers should be the same as those in monoaxial nanofibers. Therefore, the drug density relationship between core-shell nanofibers and monoaxial nanofibers should be followed,

(1) $$D_{c}=\frac{D}{Percentage\;of\;core\;layer\;mass\;in\;core-shell\;nanofiber}$$

(2) $$D_{s}=\frac{D}{Percentage\;of\;shell\;layer\;mass\;in\;core-shell\;nanofiber}$$

where D is the drug density of the monoaxial nanofiber; *D_c_* is the drug density of the core layer; and *D_s_* is the drug density of the shell layer in a core-shell nanofiber. The core layer nozzle was 30 G (inner diameter: 0.13 mm and outer diameter: 0.31 mm); the shell layer nozzle was 22 G with 0.41 mm inner nozzle diameter and 0.7 mm outer diameter, therefore, there was a channel with a 0.1 mm for the shell layer solution. After calculation, 5 μg/mL hEGF was mixed into 7 *w*/*v*% PEO solution as the core layer and 12.5 mg/mL ibuprofen was mixed into the 1:1 CCS/PEO solution as the shell layer, then the core layer and shell layer solutions were stirred for 15 min in an ice bath, respectively. The other operational conditions were the same as those for the production of monoaxial nanofibers.

### 2.3. Characterization of Nanofibers

#### 2.3.1. Morphology and Structures

The morphology of nanofibers were studied with scanning electron microscopy (SEM, JSM-IT300, JEOL, Tokyo, Japan). Prior to testing, the obtained nanofibers were coated with Au by a sputter coater (JEOL JFC-1600, Tokyo, Japan), and the diameters of the fibers were measured with ImageJ image processing software. For each type of fiber specimen, three different SEM images were taken and 100 counts of fibers were measured to calculate the mean fiber diameters and the fiber diameter distribution. Core-shell structure of the nanofibers was confirmed by transmission electron microscopy (TEM, JEM-2100 Plus, JEOL, Tokyo, Japan). For each sample, fibers were deposited onto a carbon-coated copper grid and analyzed by TEM with 200 kV.

#### 2.3.2. Viscosity

The viscosities of PEO (6 and 7 *w*/*v*%), CCS (14 *w*/*v*%), 1:1 CCS/PEO, and 2:1 CCS/PEO solutions were measured by an AR-2000ex rheometer (TA Instruments, New Castle, DE, USA) with various ratios by following the experimental procedures reported previously [41]. The test was measured at room temperature.

#### 2.3.3. Contact Angle

The contact angles of deionized water on the nanofiber scaffolds were tested through a contact angle goniometer (KURSS, DSA100, WCA, Frankfurt, Germany). A total of 15 μL of deionized water was placed on the surface of the mats and the contact angle was measured after 2 s. The test was measured at room temperature.

#### 2.3.4. Thermal Properties 

The thermal properties of fibers were tested by differential scanning calorimetric (1 DSC, 204F1, Netzsch, Selb, Germany) and thermogravimetric analysis (TGA, 209F1, Netzsch, Selb, Germany). Around 5 mg of polymer fibers were used for each sample. In the DSC test, the temperature increased from 30 °C to 250 °C with a heating rate of 10 °C min^−1^. In the TGA measurement, each sample was conducted from 30 °C to 600 °C with a heating rate of 10 °C min^−1^.

#### 2.3.5. Mechanical Tests

Mechanical tests were performed for all samples via an Instron 5943 (Instron, MA, USA) with a 50 N load cell. Each sample was twisted into a yarn with a 0.5 mm diameter and cut into 10 mm lengths. The extension rate of sample was 2 mm per minute, and the Young’s modulus and failure work were calculated from the stress–strain curve. Each type of sample was tested three times. The test was measured at room temperature.

#### 2.3.6. Characterization of Chemistry

Fourier transform infrared spectroscopy (FTIR, Vertex 70, Bruker, Karlsruhe, Germany) was used to study the chemical characteristics. All samples were tested at wavelengths in the range of 4500–400 cm^−1^ and a resolution of 1 cm^−1^ with 64 scans.

### 2.4. In Vitro Drug Release Studies

The release tests of ibuprofen loaded in the shell layer of the core-shell structured nanofibers and monoaxial nanofibers were performed by the dialysis bag (3500 Da) method with 0.1 M PBS solution (pH 7.4) as the receptor medium. The cumulative release profile of hEGF was established by measuring the amount of hEGF in the collected PBS medium by the hEGF ELIAS Kit by following the manufacturer’s instruction. The release tests of hEGF loaded in the core layer of the core-shell structured nanofibers and monoaxial nanofibers were performed by the dialysis bag (14 kDa) method with 0.1 M PBS solution (pH 7.4) as the receptor medium. 

PBS was used to mimic the wound environment and maintain wet conditions during the experiments. For each experiment, 10 mg of nanofiber scaffolds was loaded into a dialysis bag, then put into 10 mL PBS solution at 37 °C constant temperature. The samples were placed on an orbital shaker at 200 rpm. At specific intervals (0.25, 0.5, 2, 4, 12, 24, 48 h), 1 mL released medium was collected and stored at −20 °C for a later test, then replaced with 1 mL fresh PBS solution. The release profile was analyzed via ELISA at 265 nm for ibuprofen and 450 nm for hEGF, respectively. Each sample was tested three times.

### 2.5. Cell Viability Assay

Cell viability of HSF on different nanofiber mats and AquacelAg were operated according to ISO standard 10993-12 2007 via the MTT assay. Before the experiments, all samples (including core-shell nanofibers with drugs, monoaxial nanofibers with drugs, core-shell nanofibers without drugs (negative control) and AquacelAg (positive control)) were vacuum dried and UV sterilized for 30 min. To prepare the extraction solution, each specimen was placed into 10% DMEM solution with the ratio of specimen surface area and solution volume as 1.25 cm^2^/mL, then incubated in an aseptic incubator for 24 h at 37 °C. Using HSF in a 96-well plate, a 100 μL HSF cell suspension (0.5 × 10^4^ count) was plated into every well. After 24 h incubation in a 5% CO_2_ incubator, HSF was seeded into the extraction solution and incubated for 12 h, 24 h, and 48 h. Then, the wells were washed with PBS twice, and 200 μL MTT (5 mg/mL) was dropped into the wells and incubated for 24 h. After incubation, 150 μL DMSO was dropped into each well and put the well-plate on a shaker with low shaking for 10 min. Optical density of the viable cells was read by ELISA at an OD of 570 nm. The cell viability was calculated by:

(3) $$\mathrm{cell}\;\mathrm{viability}\;\%=\frac{\mathrm{mean}\;\mathrm{optical}\;\mathrm{density}}{\mathrm{control}\;\mathrm{optical}\;\mathrm{density}}\times 100$$

### 2.6. Antibacterial Evaluation 

To evaluate the antibacterial activities of the nanofibrous scaffolds via centrifugal spinning, four different scaffolds were used including core-shell nanofibers loaded with hEGF in the core layer and ibuprofen in the shell layer (named as core-shell nanofiber with drugs), and monoaxial nanofibers loaded with hEGF and ibuprofen (named as monoaxial nanofiber with drugs). AquacelAg and core-shell nanofibers without drugs were chosen as the positive control and the negative control, respectively. *Escherichia coli* (*E. coli*), *Pseudomonas aeruginosa* (*P. aeruginosa*), and *Staphylococcus aureus* (*S. aureus*) were used to assess the antibacterial activities of nanofibrous samples and AquacelAg. Before the experiments, all samples were vacuum dried and UV sterilized for 30 min. All of the prepared bacterial strains were incubated in Luria-Bertani medium with an orbital shaker at 37 °C/220 rpm, until the OD value at 600 nm reached 0.6 (bacterial concentration was about 1 × 10^8^ CFU/mL). Each bacterial suspension was diluted 100 times. Then, 15 mL of the diluted bacterial suspension was spread onto a Petri dish (9 cm diameter). The disk-shaped (1 cm diameter) nanofibrous mats and AquacelAg mats were placed onto the corresponding bacterial Petri dish and incubated for 24 h at 37 °C. Then, the inhibition zone was photographed and measured. The experiments were repeated three times and operated in an aseptic environment. 

### 2.7. Statistical Analysis

Statistical analysis of all data was calculated as the mean + standard deviation (SD), and performed using one-way ANOVA. A probability value (*p*) < 0.05 was considered as statistically significant.

## 3. Results and Discussion

### 3.1. Design of Co-Axial Centrifugal Spinning Device for Producing Core-Shell Nanofiber

Centrifugal spinning is a cost-effective method to fabricate nanofibers, which utilizes centrifugal force to drag the solution jet, then the extended jet forms the nanofiber after solvent evaporation. It is an alternative method to overcome the drawbacks of electrospinning [42]. Core-shell nanofibers could also be produced via centrifugal spinning [18], however, the core-shell structure of the nanofibers via centrifugal spinning is yet to be confirmed and demonstrated by electron microscope analysis.

**Figure 1:**
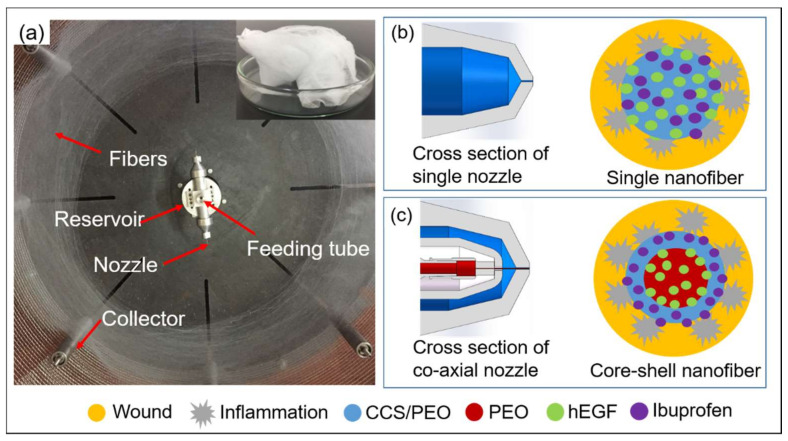
(**a**) Centrifugal spinning machine and the collected 3D structured nanofibers; schematic diagrams of (**b**) monoaxial nanofibers with drugs and (**c**) core-shell nanofibers with drugs in a wound environment.

A centrifugal spinning device was designed for the fabrication of monoaxial structured nanofibers [41] (Figure 1a), however, it was difficult to control the drug release in the monoaxial nanofibers produced as the bio-active drug (such as hEGF) mixed in the monoaxial nanofibers might rapidly lose bio-activity in a wound environment without protection (Figure 1b). The centrifugal spinning machine was further modified by replacing the monoaxial liquid reservoir and monoaxial nozzle with co-axial liquid reservoirs and co-axial nozzles, where two solutions can be fed into the core and shell liquid channels, respectively. The cross section of the partial engineering drawing via Solidworks software is shown in Figure 1c. Therefore, the hEGF in the core layer could be protected by a shell layer with antibacterial properties and released at the phase of proliferation. The mechanical structure of the co-axial nozzle in centrifugal spinning is similar to that in co-axial electrospinning. In order to produce a stable co-axial jet in co-axial electrospinning, Szentivanyi et al. [11] reported that the flow rate ratio of the core and shell layers should be between 1:3 to 1:6. It was assumed that the flow rate ratio of the core and shell layers in co-axial centrifugal spinning should also be within an appropriate range to form a stable co-axial liquid jet. The flow rates of the core and shell layers can be changed by controlling the size of the co-axial nozzles. 

Compared with the reservoirs for monoaxial nanofibers, the mechanical structure of co-axial reservoirs for core-shell structured nanofibers was more complex. The reservoir for the core solution must be smaller than the reservoir for the shell solution for the core and shell layers to form co-axial channels. The engineering drawing of the key parts in co-axial centrifugal spinning is shown in Figure S1. The assembled co-axial reservoirs are shown in Figure S2.

### 3.2. Characterizations of Nanofibers

#### 3.2.1. Investigation of Parameter Control for Producing Core-Shell Nanofibers

Centrifugal spinning method utilizes the centrifugal force to produce nanofibers. The parameters of the centrifugal spinning system can be divided into solution intrinsic properties and operational conditions [18]. Solution intrinsic properties are solution properties such as viscosity, concentration, molecular weight, surface tension, and solvent type. However, for a certain polymer material, the solution viscosity significantly affected the nanofiber formation [43,44]. The operational conditions included nozzle diameter, rotational speed, nozzle length, collection distance, and temperature, but nozzle diameter and rotational speed were the key parameters for nanofiber formation [16,43,45].

In this study, viscosity, co-axial nozzle diameters, and rotational speed were studied for the fabrication of the core-shell nanofibers (Table S1). No fiber was formed when the outer diameter of the shell layer nozzle was at 0.51 mm (samples A and B) or rotational speed at 4000 rpm (samples C and D) and only a few fibers could be collected with rotational speed at 5000 rpm (samples E and F). However, the fibers could be stably collected when the rotational speed was 4500 rpm and the outer diameter of shell nozzle was 0.41 mm (samples G–J). No fiber formed in samples A and B possibly because the nozzle diameter of the shell layer was too big to make the solution jet at the jet-initiating stage without enough time for thinning at the jet-extension stage, and formed solid fibers after the solvent evaporated. In addition, many jets dropping on the ground might also be because the bulky solution jet was too heavy and the gravity pulled the jet down before it got on the collector (Figure S3). When the rotational speed was 4000 rpm (samples C and D), the fibers also did not form, and a very highly viscous solution attached on the outside wall of the rotational reservoir (Figure S4). This might be attributed to the Re and We of the solution jet that were too small to get rid of the elasticity effect, and the solvent evaporation time was too short to form a solid fiber. When the rotational speed was 5000 rpm (samples E and F), only a few fibers could be collected, as shown in Figure S5. This might be because of the high rotational speed and large stretch force to make the jet break up earlier, so the formed fiber was not long enough, and a large number of formed fibers flew away in the turbulence air field and could not be collected on the collector. Samples G–J obtained continuously stable fibers, so the study and analysis concentrated on these four samples.

**Figure 2:**
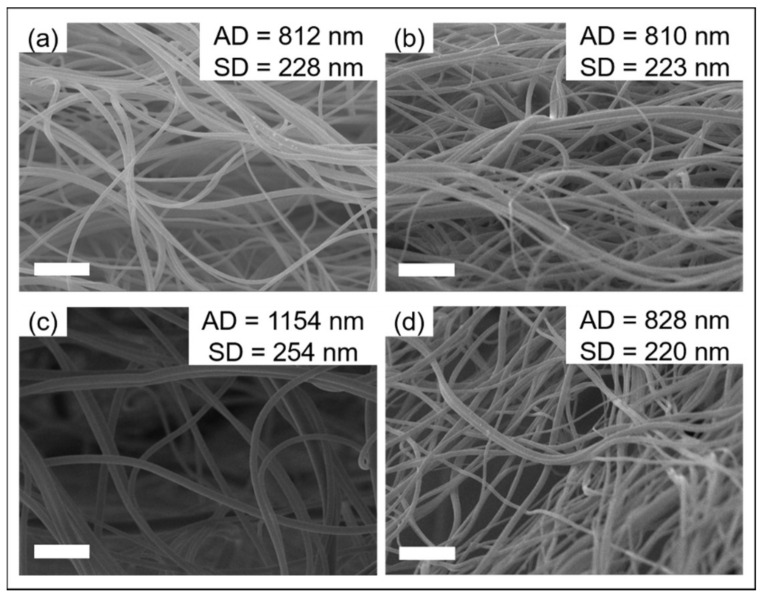
SEM images of nanofibers via co-axial centrifugal spinning. (**a**) Sample G: 7 *w*/*v*% PEO and 2:1 CCS/PEO; (**b**) Sample H: 6 *w*/*v*% PEO and 2:1 CCS/PEO; (**c**) Sample I: 7 *w*/*v*% PEO and 1:1 CCS/PEO; (**d**) Sample J: 6 *w*/*v*% PEO and 1:1 CCS/PEO. Scale bar: 10 μm.

**Figure 3:**
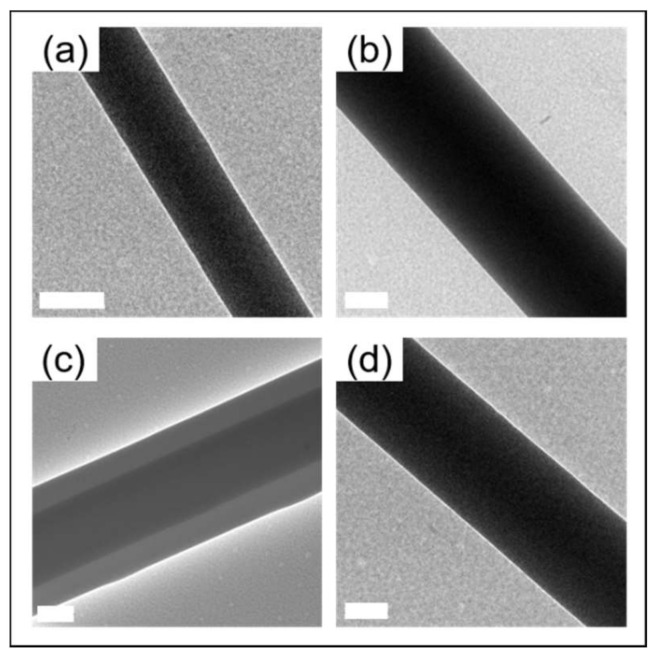
TEM images of nanofibers via co-axial centrifugal spinning. (**a**) Sample G: 7 *w*/*v*% PEO and 2:1 CCS/PEO; (**b**) Sample H: 6 *w*/*v*% PEO and 2:1 CCS/PEO; (**c**) Sample I: 7 *w*/*v*% PEO and 1:1 CCS/PEO; (**d**) Sample J: 6 *w*/*v*% PEO and 1:1 CCS/PEO. Scale bar: 200 nm.

Figure 2 and Figure 3 show the morphologies and structures of the nanofibers (Samples G–J in Table S1) via a co-axial centrifugal spinning device. The morphologies of the produced nanofibers were smooth and uniform. However, the core-shell structured nanofiber was only captured when the solutions in the core and shell layers were 7 *w*/*v*% PEO and 1:1 CCS/PEO (Sample I) and the average diameter of Sample I was higher than the average diameter of other samples. 

The viscosity of polymer solutions for both the core and shell layers will determine whether a core-shell fiber can be formed. The polymer solution is a non-Newtonian liquid and the fiber formation can be described by dimensionless numbers [46]. The difference in the viscosity of the core and shell layers could significantly affect the formation of core-shell nanofibers. Jet-extension is the most critical stage for the formation of core-shell nanofibers as the jet behaviors of the core and shell layers were affected by various effects including the centrifugal force, inertia effect, viscous effect, and elasticity effect [44]. The difference in parameters between samples G–J were only the PEO solution concentrations or the ratios of CCS to PEO. This illustrates that the viscous force and elasticity force, which were changed with solution intrinsic properties, were highly related to the formation of the core-shell nanofiber in this study. 

**Figure 4:**
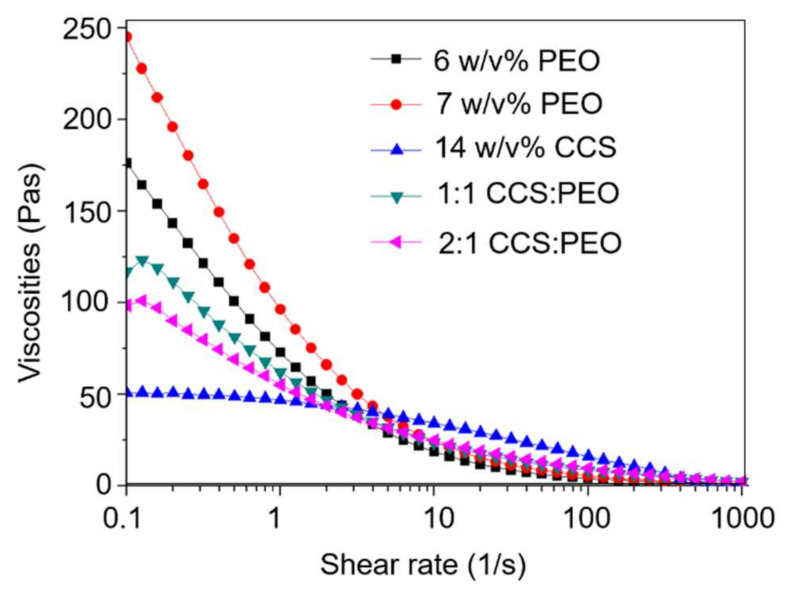
Dynamic viscosity of the different solutions via a rheometer.

In addition, the dynamic viscosities of various solutions in the core and shell layers were varied by changing the concentration of PEO or the ratio of CCS/PEO (Figure 4). Besides, the curvature radius of the polymer jet in the core and shell layers can be described by Reynolds (Re) and Weber (We) numbers [46]:

(4) $$Re=\rho v_{0}r_{0}/\eta_{0}$$

(5) $$We=\rho v_{0}^{2}r_{0}/\gamma$$

where ρ is the density; $v_{0}$ is the jet velocity; $r_{0}$ is the jet radius; $\eta_{0}$ is the solution viscosity; and γ is the solution surface tension. The jet velocity is not only influenced by operational conditions (such as rotational speed and nozzle diameter), but also influenced by viscosities [45]. Therefore, the solution intrinsic properties significantly influence Re and We numbers, which influences the curvature radius of the jet. When the solutions in the core and shell layers are 7 *w*/*v*% PEO and 1:1 CCS/PEO (Sample I condition), the curvature radiuses of the core layer jet and shell layer jet could be the same, so the two layers still remain at the co-axial jet at the jet-extension stage. Therefore, the core-shell structured jet remained at the solvent evaporation stage and the core-shell structured nanofiber was formed after core-shell jet solidification. The core-shell structured nanofiber could not be characterized by TEM in three other samples. This might be because the Re and We numbers of the core layer and shell layer jets were different, resulting in these two jets not sharing the same curvature radius, hence the core layer might get rid of the encapsulation from the shell layer or the solutions in the core and shell layers were mixed at the jet-extension stage. 

#### 3.2.2. Thermal Properties 

**Figure 5:**
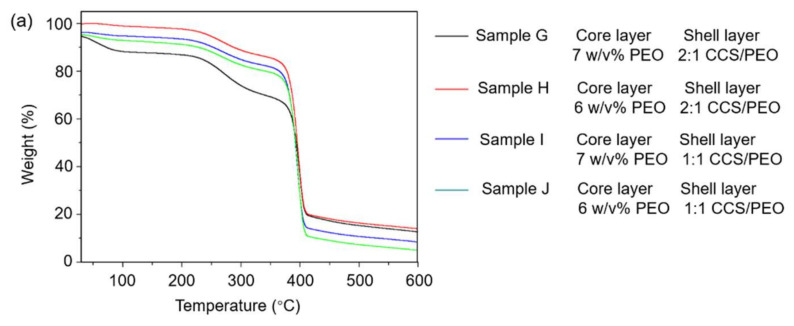

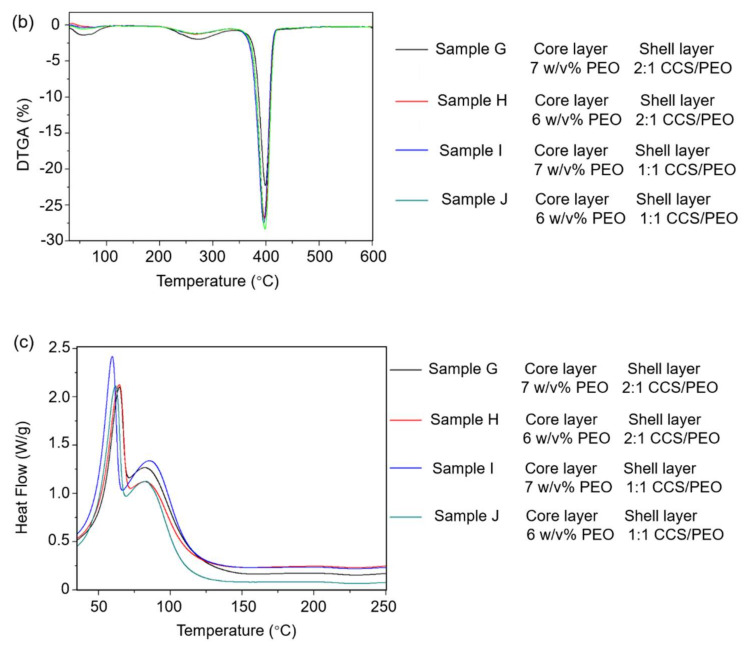
(**a**) TGA, (**b**) DTGA, and (**c**) DSC of nanofibers via co-axial needle centrifugal spinning.

Figure 5 shows the thermal properties of the nanofiber scaffolds via the co-axial centrifugal spinning method using thermogravimetry (TGA) and differential scanning calorimetry (DSC). The curves of the TGA and differential thermogravimetric analyses (DTGA) showed that the weight loss that occurred below 100 °C should be the moisture present in the nanofiber scaffolds [35,47]. In addition, a higher weight loss for sample G below 100 °C might be because there was more humidity in the scaffold. In the range of 100–200 °C, all samples were stable and there was hardly any weight loss. After 200 °C, these scaffolds started losing weight again, peaked at 270 °C, and slowed down around 340 °C. This might be due to the nitrogenization of the CCS in the fiber scaffolds. The largest weight loss occurred between 340–430 °C and peaked at around 400 °C, which might be due to the carbonization of PEO and CCS in the scaffolds [48]. When the temperature was over 430 °C, the weight of these samples was stable, which indicated that the degradation was finished. Finally, the higher content of CCS led to a larger weight residue due to the carbon element content in CCS being higher than PEO. 

DSC curves of the four samples via co-axial centrifugal spinning were similar (Figure 5c). There were two endothermic peaks in each sample, the first at around 65 °C and the second at around 85 °C, indicating a two-stage crystallization model [49]: an uncompleted crystallization at lower temperature and a secondary crystallization at higher temperature. This result illustrated that the covalent bond between the two phases of the block copolymer prevented them from partial component separation [50]. As observed in our previous study, the DSC curves of nanofiber via monoaxial needle centrifugal spinning had only one endothermic peak at around 65 °C, regardless of the ratio of CCS to PEO [41]. Additionally, many studies have reported that there were two endothermic peaks in DSC curves of the core-shell structure [51,52,53,54,55,56]. Therefore, it could demonstrate that all of the four samples via co-axial centrifugal spinning had core-shell structured nanofibers.

On the other hand, the endothermic peaks of sample I were higher than the others, which indicated that the covalent bonds between the two phases in sample I were more than the other samples. This might be attributed to more phases of the block copolymer existing in sample I than in the other samples, which further explains that core-shell structures formed in sample I compared with the other samples. 

#### 3.2.3. Mechanical Properties 

**Figure 6:**
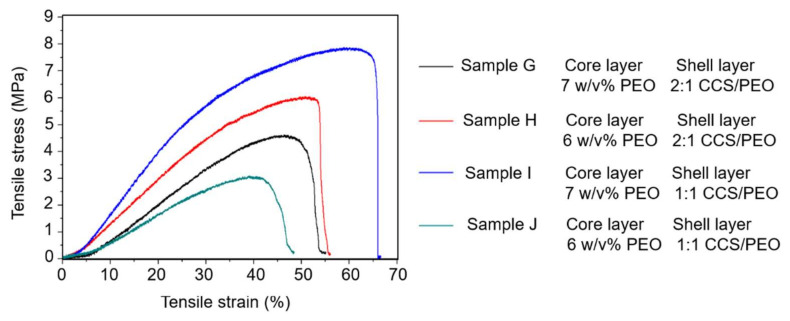
Mechanical test for nanofibers via co-axial needle centrifugal spinning.

The mechanical properties of samples G–J in Table S1 is shown in Figure 6. Sample J had the lowest tensile strain of these four samples with around 45% while sample I had the highest tensile strain of over 65%. The tensile stress of these four samples was between 3.1–7.8 MPa, which could be used as tissue scaffolds for cell migration and proliferation due to the tensile stress of ECM being in the range of 0.8–18 MPa [57,58]. Therefore, these four nanofiber yarns produced from co-axial centrifugal spinning were sufficiently strong for cell culture [59]. 

Mechanical properties of the nanofibers are strongly influenced by many factors including the chemical structure of polymers and the physical properties of fibers (structure, morphology, and diameter). As a previous study demonstrated that a higher CCS component led to better mechanical properties [41], the mechanical properties of samples G and H should be better than samples I and J. However, the test on mechanical properties showed that sample I had the largest tensile stress and longest tensile strain. This might be attributed to the core-shell structures in sample I. In the core-shell structured nanofiber, there were some molecular interactions (such as Van der Waals interaction and covalent bond) between the core layer and shell layer that will improve the mechanical properties of the fibers. Similar results with improved mechanical properties in core-shell structured nanofibers have been demonstrated in other studies [57,60]. 

#### 3.2.4. Fourier Transform Infrared Spectroscopy (FTIR)

**Figure 7:**
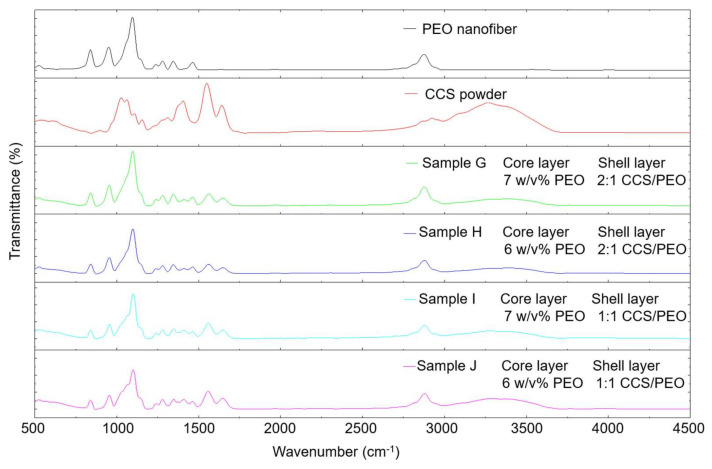
FTIR spectra of the pure PEO, pure CCS, and composite nanofibers via co-axial centrifugal spinning.

The functional groups in the produced nanofibers (samples G–J) via co-axial centrifugal spinning as well as the pure PEO and pure CCS were characterized by FTIR (Figure 7). The fingerprint range at 750–1500 cm^−1^ can be attributed to molecular bending vibrations [61]. The peak at 840 cm^−1^ corresponded to the C–O stretching [62] and the peak at 950 cm^−1^ was due to the vibration of the ether group [36]. The peaks at 1026 and 1060 cm^−1^ in CCS can be attributed to the C–O stretching vibrations of the pyranose ring [63]. These two peaks were not obvious in the CCS/PEO composite because the absorption values were weak and covered by the sharp peak at 1100 cm^−1^. Similar result were also demonstrated in the study by Lin et al. [64]. The peak at 1100 cm^−1^ can be attributed to the C–O–C stretching vibrations [35,47]. The peaks at 1342 cm^−1^ corresponded to O–H deformation mode [65]. Two peaks at 1560 and 1649 cm^−1^ were attributed to amide groups [47,66]. The peak at 2879 cm^−1^ was due to the CH_2_ stretching [67]. A broad bond at 3200–3400 cm^−1^ was detected due to N–H and O–H stretching of the polysaccharide molecules [66]. All characterized peaks in PEO and CCS were shown in the CCS/PEO composite nanofibers via co-axial centrifugal spinning. This demonstrates that there was no chemical reaction during the production of core-shell nanofibers via co-axial centrifugal spinning.

#### 3.2.5. Contact Angle Studies

**Figure 8:**
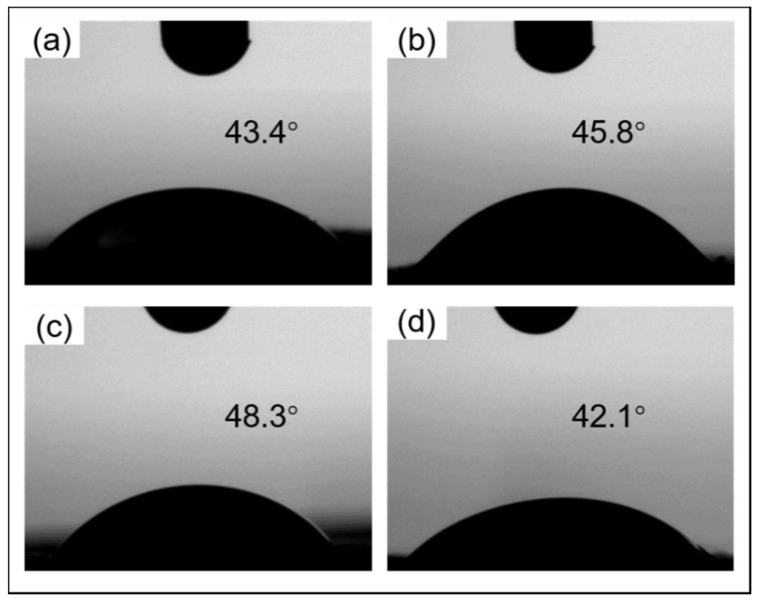
Contact angle images of nanofibers via co-axial needle centrifugal spinning. Core and shell layers in these four samples: (**a**) Sample G was 7 *w*/*v*% PEO and 2:1 CCS/PEO; (**b**) Sample H was 6 *w*/*v*% PEO and 2:1 CCS/PEO; (**c**) Sample I was 7 *w*/*v*% PEO and 1:1 CCS/PEO; and (**d**) Sample J is 6 *w*/*v*% PEO and 1:1 CCS/PEO.

The contact angle of the nanofibers is also influenced by the chemical composition and fiber morphology. The CCS percentage in samples G and H was higher than that in samples I and J, therefore, samples I and J should be more hydrophilic than samples G and H [36,68]. However, Figure 8 indicates that sample I was more hydrophobic (a larger contact angle than other composite nanofiber mats). This might be because the core-shell structured nanofiber is more hydrophobic than the monoaxial structured nanofiber [69]. 

### 3.3. In Vitro Drug Release Profile

**Figure 9:**
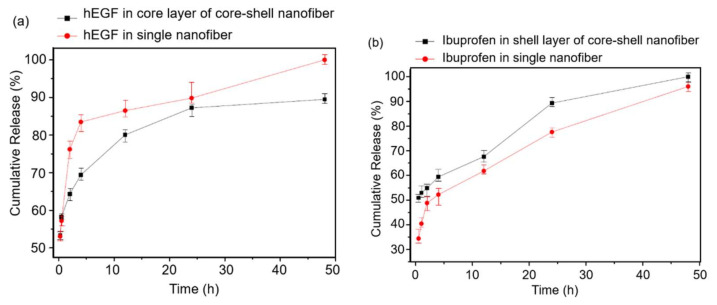
Drug release profiles of (**a**) hEGF and (**b**) ibuprofen in the core-shell and monoaxial nanofibers.

The drug release mechanisms in nanofibers mainly depend on drug diffusion, polymer nanofiber biodegradation, and nanofiber erosion [70]. The hEGF release profiles in the core layer of the core-shell nanofibers and monoaxial nanofibers are shown in Figure 9a. On the whole, the hEGF release rate in the core layer of core-shell nanofibers was lower than that in monoaxial nanofibers. A dramatical drug burst occurred in the monoaxial nanofibers at the first 2 h, with over 75% of the total hEGF released, then, the rest of the hEGF gradually and fully released in the end. Besides, the hEGF release rate in the core layer of the core-shell nanofiber was moderate. This might mainly be attributed to the hEGF being embedded in the core layer, which was encapsulated by the shell layer, and the hEGF release rate was controlled by the encapsulation. In addition, the drug release rate was also influenced and controlled by the fiber diameter. The average diameters of core-shell nanofibers and monoaxial nanofibers were 1154 nm and 481 nm, respectively. Therefore, the polymer erosion rate of the core-shell nanofibers should be lower than monoaxial nanofibers and the core layer polymer erosion rate was lower than the shell layer as the surface erosion occurred initially on the shell layer.

The ibuprofen release profiles in the shell layer of core-shell nanofibers and monoaxial nanofibers are shown in Figure 9b. At the beginning, the release rate of ibuprofen in the core-shell nanofibers was over 50%, but only around 35% in monoaxial nanofibers. Even though there was a little burst in monoaxial nanofibers at 2 h, the release rate was still lower than the core-shell nanofibers during the whole period. This result could be explained by the different initial position of ibuprofen in the core-shell and monoaxial nanofibers, and the different polymer erosion mechanism. As previously discussed, the polymer solution flow rate of the core layer to shell layer was 1:4.3. In order to make sure that the ibuprofen quantity in the core-shell nanofibers and monoaxial nanofibers was equal, when the weight of the core-shell nanofibers and monoaxial nanofibers were the same, the density of ibuprofen in the shell layer of the core-shell nanofibers should be around 1.25 times that in the monoaxial nanofiber. The ibuprofen diffusion rate in core-shell nanofibers should be higher than monoaxial nanofibers due to the drug diffusion mechanism [71]. Additionally, the shell layer was the surface of the core-shell nanofibers, so the erosion of shell layer should be faster than the erosion of the monoaxial nanofiber as the surface erosion rate was greater than the bulk erosion rate [72]. Therefore, the ibuprofen release rate in the shell layer of the core-shell nanofibers was higher than that in the monoaxial nanofibers. 

### 3.4. Cell Viability Assessment

**Figure 10:**
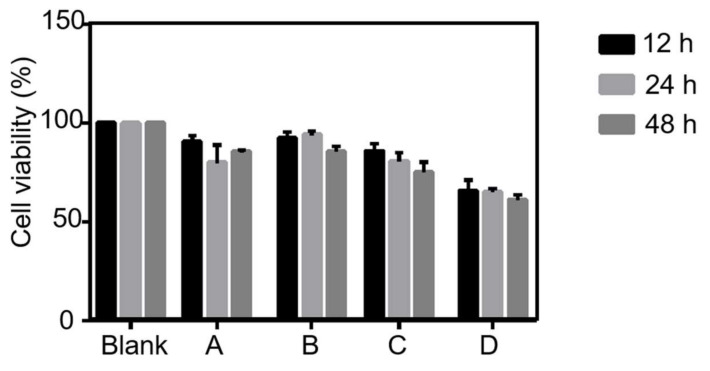
Cell viability determined by the MTT assay for the mats. (A) core-shell nanofiber mat with drugs, (B) monoaxial nanofiber mat with drugs, (C) AquacelAg, and (D) core-shell nanofiber mat without drugs. Data represent mean ± SD.

The viability of HSF cells on the produced core-shell nanofiber mat with hEGF, monoaxial nanofiber mat with hEGF, and core-shell nanofiber mat without hEGF (negative control) as well as the commercial wound dressing, AquacelAg (positive control), were investigated by the MTT assay, as shown in Figure 10. It was observed that both core-shell and monoaxial nanofiber mats with hEGF exhibited a higher cell viability than the others; AquacelAg had a moderate cell viability, and the nanofiber mat without hEGF represented the lowest cell viability during the cell culture period. This indicates that hEGF poses a significant impact on the HSF cell proliferation, and the efficacy was better than the commercial wound dressing. 

Notably, the cell viability in the nanofiber mats with hEGF tended to fluctuate from time to time, while the cell viability in the positive and negative control always decreased. At 12 h, the cell in the core-shell nanofiber mat with hEGF and the monoaxial nanofiber mat with hEGF had a similar viability of 91% and 92%, respectively. At 24 h, the cell viability in core-shell nanofiber with hEGF decreased to 80%, but the value at monoaxial nanofibers with hEGF increased to 94%. This might be because the release rate of hEGF in the core layer of the core-shell nanofibers was slower than that in the monoaxial nanofibers, and the reacted hEGF in the medium with monoaxial nanofibers was larger than that in the medium with core-shell nanofibers at the period. Therefore, the cell viability in the monoaxial nanofiber was better than in the core-shell nanofiber. However, the cell viability in the core-shell nanofiber with hEGF increased to 86% and the cell viability in the monoaxial nanofiber with hEGF decreased to 86% at 48 h. This might be because the hEGF in the core layer of the core-shell nanofiber sufficiently released and reacted after 24 h, while the hEGF in the monoaxial nanofiber burst released at the beginning of a few hours. Therefore, HSF cells in the core-shell nanofiber were more active as hEGF was stably released, and the cell viability in the monoaxial nanofiber decreased after hEGF burst release at the initial time. The final cell viability of the core-shell nanofiber and monoaxial nanofiber was the same.

### 3.5. In Vitro Antibacterial Assessment

Chitosan is an attractive biodegradable and biocompatible polymer for wound healing materials due to its antibacterial and antifungal properties [73]. However, the antibacterial abilities of chitosan are not strong enough to inhibit bacteria growth [74]. In this study, ibuprofen was embedded into the CCS composite nanofibers to improve the antibacterial properties of nanofibrous mats because ibuprofen is a widely used analgesic drug with an anti-inflammatory property that might improve the inhibition ability in wound healing [10]. The antibacterial abilities of different mats were assessed by measuring the diameter of the inhibition zone.

**Figure 11:**
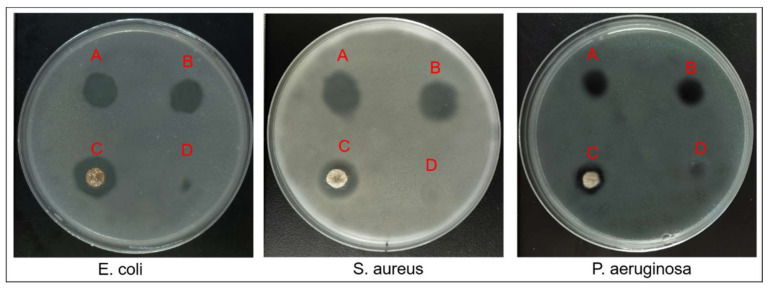
Inhibition effect of (A) core-shell nanofiber with drugs, (B) monoaxial nanofiber with drugs, (C) AquacelAg, and (D) core-shell nanofiber without drugs against *E. coli*, *S. aureus*, and *P. aeruginosa*, respectively.

The growth inhibition zones of bacteria (*E. coli*, *S. aureus* and *P. aeruginosa*) after 24 h of incubation with nanofiber mats and AquacelAg are shown in Figure 11 and Table 1. It was observed that the monoaxial nanofiber with drugs, the core-shell nanofiber with drugs, and AquacelAg had the similar diameter of inhibition zone in corresponding bacteria Petri dishes. However, the core-shell nanofiber without drugs had few effects on *E. coli* with only a 3.6 mm diameter of inhibition zone, and almost no effect on *S. aureus* and *P. aeruginosa* due to the zone still being occupied by some bacteria (light color zone). This indicates that the CCS composite mat had weak antibacterial properties without ibuprofen.

## 4. Conclusions

In addition, the core-shell nanofiber with drugs and monoaxial nanofiber with drugs had the similar antibacterial properties to AquacelAg because both CCS composite mats were loaded with ibuprofen, which is an effective drug for the inhibition of bacteria [31]. After 24 h, the nanofiber mats of the core-shell nanofiber with drugs and monoaxial nanofiber with drugs all dissolved into solution, while AquacelAg had some undissolved mats even though it was hydrophilic. In wound healing applications, part of the dressing embeds into or adheres on the repairing tissues during wound healing. However, the repairing tissues very likely suffer secondary damage and secondary contamination from the conventional nondegradable wound dressing during dressing change [75]. Therefore, the fabricated CCS composite nanofibrous mats with drugs might be more suitable for wound healing compared with the positive control (AquacelAg) because the fibers embedded into or adhered on the repairing tissues had degraded into tissues.

In this study, a co-axial centrifugal spinning device was developed to fabricate core-shell nanofibers. After investigation, core-shell structural nanofibers via co-axial centrifugal spinning could be stably produced, and the mechanical property of the core-shell nanofiber was better than the monoaxial nanofiber. Besides, in vitro experiments demonstrated the advantages of the produced core-shell nanofiber for potential wound healing application. Multiple drugs could be loaded into the different layers of the core-shell nanofiber to control the drug release rate in different phases for specific drug delivery and improve the drug efficiency during the wound healing process. The production rate of co-axial centrifugal spinning is much higher than traditional co-axial electrospinning, showing the potential of commercialization.

## Figures and Tables

**Table 1:** Average inhibition sizes of four different samples against *E. coli*, *S. aureus*, and *P. aeruginosa*.

Bacteria	Inhibition Size of A (mm)	Inhibition Size of B (mm)	Inhibition Size of C (mm)	Inhibition Size of D (mm)
*E. coli*	12.5	12.1	3.6	14.9
*S. aureus*	16.0	15.8	-	15.4
*P. aeruginosa*	10.7	10.1	-	11.2

## Data Availability

Data is contained within the article or supplementary material.

## Supplementary Materials

The following are available online at https://www.mdpi.com/article/10.3390/nano11061546/s1, Figure S1: The engineering drawing of the key parts in co-axial centrifugal spinning: core layer reservoir (a), core nozzle channel (b), core nozzle cover (c), shell layer reservoir (d), shell nozzle (e) and flange (f), Figure S2: The designed and assembled co-axial reservoirs for fabrication of core-shell nanofiber, Figure S3: Image and schematic of polymer jets drop on ground, Figure S4: Image and schematic of polymer jets collect on rotational reservoir, Figure S5: Image and schematic of only few continuous fibers collect on collector wall, Table S1: Fabrication of nanofibers via co-axial centrifugal spinning with various parameter.
