# Supplementary material for: Multi-Functional Core-Shell Nanofibers for Wound Healing
Source: Nanomaterials (Basel). 2021 Jun 11;11(6):1546. doi: 10.3390/nano11061546 (PMC8230886; doi:10.3390/nano11061546)
Supplement: Supplementary file 1 [file nanomaterials-11-01546-s001.zip › nanomaterials-1233989-supplementary.pdf]

(a)

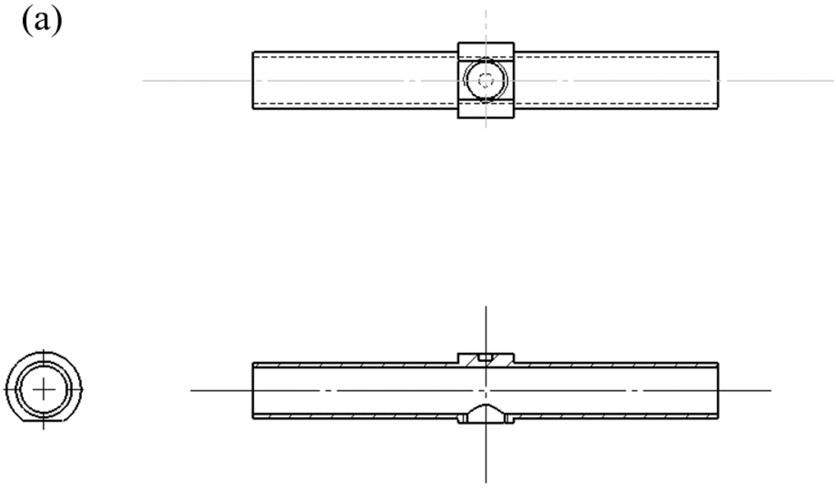

(b)

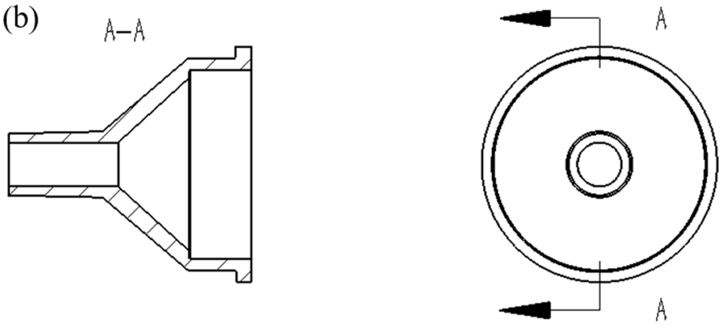

(c)

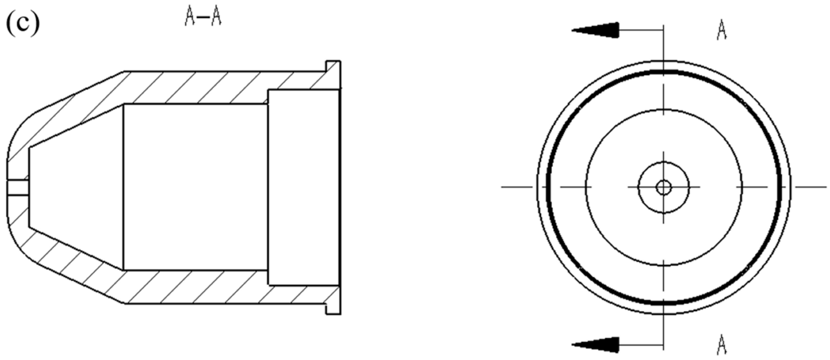

(d)

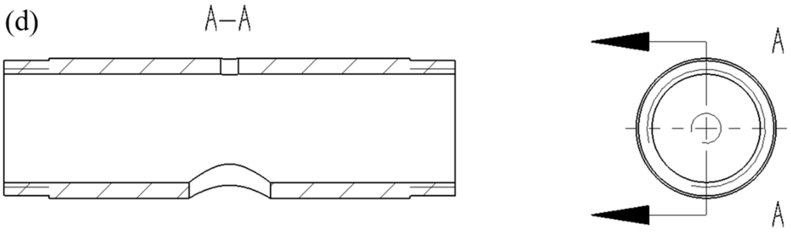

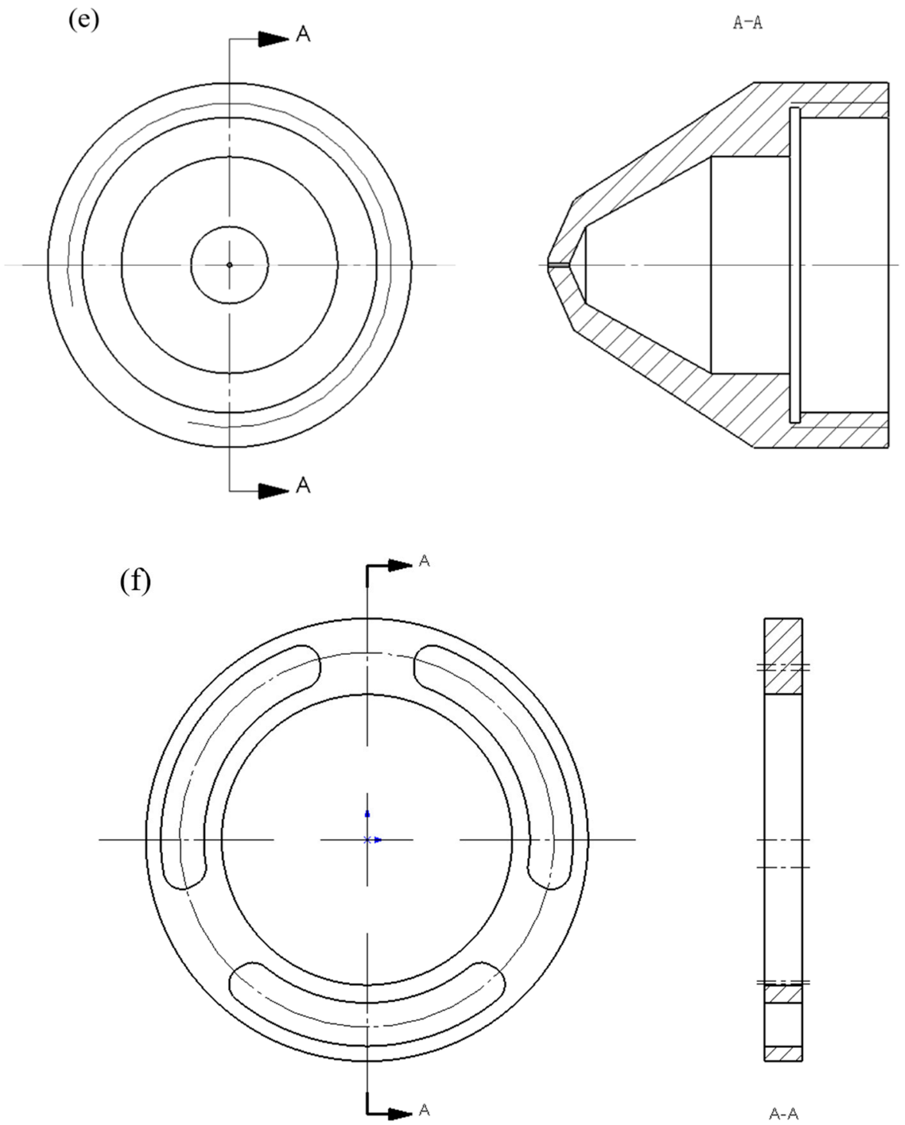

Figure S1. The engineering drawing of the key parts in co-axial centrifugal spinning: core layer reservoir (a), core nozzle channel (b), core nozzle cover (c), shell layer reservoir (d), shell nozzle (e) and flange (f).

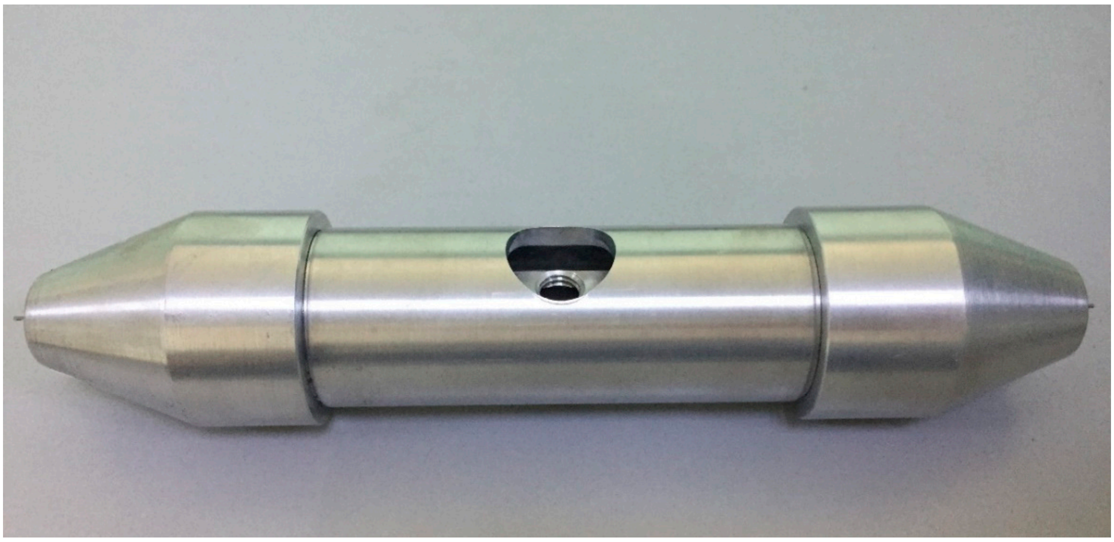

Figure S2. The designed and assembled co-axial reservoirs for fabrication of core-shell nanofiber.

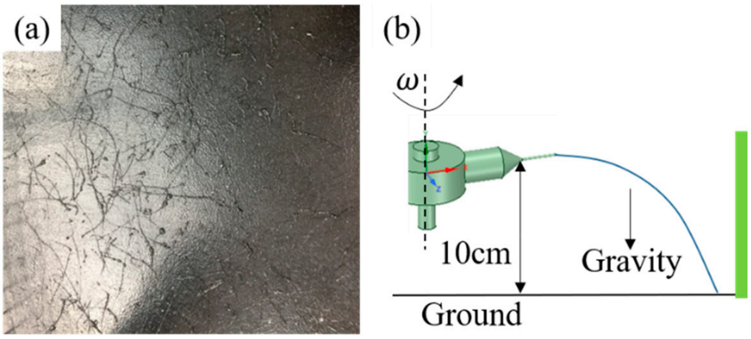

Figure S3. Image and schematic of polymer jets drop on ground.

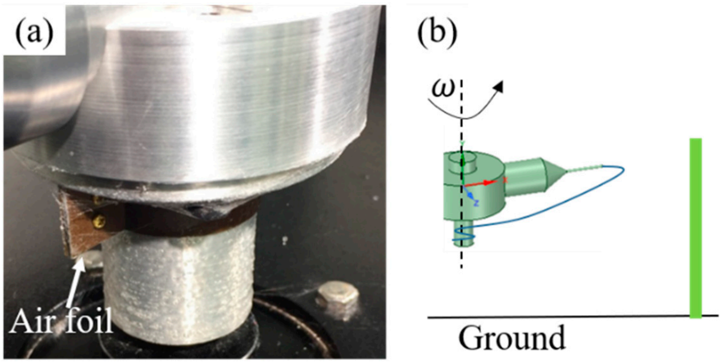

Figure S4. Image and schematic of polymer jets collect on rotational reservoir.

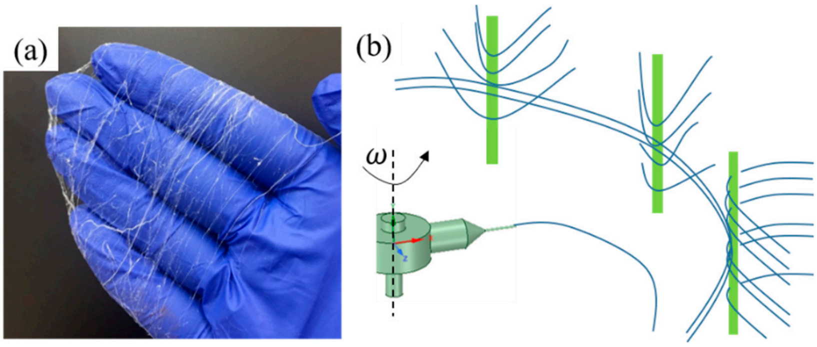

Figure S5. Image and schematic of only few continuous fibers collect on collector wall.

Table S1. Fabrication of nanofibers via co-axial centrifugal spinning with various parameter.

Sample	Rotational speed (rpm)	Core nozzle size (mm)		Shell nozzle size (mm)		Flow ratio of core to shell	Core layer solution	Shell layer solution	Fiber diameter range (nm)	Comments
		Inner diameter	Outer diameter	Inner diameter	Outer diameter					
A	4500	0.13	0.31	0.51	0.81	1:9.7	7 w/v% PEO	2:1 CCS/PEO	-	No fiber forms
B	4500	0.17	0.35	0.51	0.81	1:4.7	6 w/v% PEO	2:1 CCS/PEO	-	No fiber forms
C	4000	0.08	0.2	0.41	0.71	1:20	7 w/v% PEO	1:1 CCS/PEO	-	No fiber forms
D	4000	0.08	0.2	0.41	0.71	1:20	6 w/v% PEO	1:1 CCS/PEO	-	No fiber forms
E	5000	0.13	0.31	0.41	0.71	1:4.3	7 w/v% PEO	2:1 CCS/PEO	-	Only few fibers form*
F	5000	0.13	0.31	0.41	0.71	1:4.3	6 w/v% PEO	2:1 CCS/PEO	-	Only few fibers form*
G	4500	0.13	0.31	0.41	0.71	1:4.3	7 w/v% PEO	2:1 CCS/PEO	406-1824	-
H	4500	0.13	0.31	0.41	0.71	1:4.3	6 w/v% PEO	2:1 CCS/PEO	495-1604	-
I	4500	0.13	0.31	0.41	0.71	1:4.3	7 w/v% PEO	1:1 CCS/PEO	689-1951	-
J	4500	0.13	0.31	0.41	0.71	1:4.3	6 w/v% PEO	1:1 CCS/PEO	437-1839	-

Remarks: Sample E and F formed only few fibers, and large number of polymer solutions were waste. Therefore, it's meaningless to test these two samples.
